# Supplementary material for: Transcriptome Profiling Reveals Key Regulatory Networks for Age–Dependent Vernalization in Welsh Onion (Allium fistulosum L.)
Source: Int J Mol Sci. 2024 Dec 7;25(23):13159. doi: 10.3390/ijms252313159 (PMC11642685; doi:10.3390/ijms252313159)
Supplement: Supplementary file 1 [file ijms-25-13159-s001.zip › ijms-3202535-supplementary.pdf]

Table S1. Sequencing data statistics of all processed samples in Group A.

Sample	Clean Reads	GC Content	Q30	Mapped Reads
N5V0-1	47,362,478	43.56%	97.94%	96.63%
N5V0-2	45,322,958	43.71%	97.98%	96.55%
N5V0-3	45,840,830	43.49%	97.70%	96.84%
N5V3-1	44,520,814	43.49%	97.97%	96.81%
N5V3-2	46,503,276	43.66%	98.00%	96.98%
N5V3-3	44,369,540	44.16%	97.94%	97.01%
N8V0-1	45,578,726	43.24%	97.58%	96.56%
N8V0-2	46,707,248	43.68%	97.72%	96.88%
N8V0-3	45,985,268	43.23%	97.96%	96.64%
N8V3-1	52,725,024	44.12%	95.32%	95.00%
N8V3-2	45,602,124	43.59%	98.01%	96.81%
N8V3-3	46,114,030	43.61%	98.00%	96.58%
N8V6-1	44,529,068	43.76%	98.03%	97.06%
N8V6-2	45,771,696	43.23%	97.94%	96.93%
N8V6-3	45,672,948	43.30%	97.92%	96.87%
N11V0-1	47,770,646	43.72%	97.56%	96.66%
N11V0-2	44,474,796	43.59%	98.06%	96.65%
N11V0-3	47,957,476	43.33%	97.93%	96.60%
N14V0-1	47,040,032	43.72%	97.97%	96.88%
N14V0-2	46,213,774	43.18%	98.06%	96.67%
N14V0-3	45,560,662	43.72%	97.99%	96.91%
Total	971,623,414			

Table S2. Sequencing data statistics of all processed samples in Group B.

Sample	Clean Reads	GC Content	Q30	Mapped Reads
N4V0-1	47,132,006	44.01%	97.35%	95.80%
N4V0-2	47,581,028	43.87%	97.00%	95.63%
N4V0-3	47,936,456	44.34%	97.41%	95.86%
N4V4-1	43,422,628	44.51%	98.10%	97.40%
N4V4-2	47,328,306	44.39%	98.05%	97.32%
N4V4-3	44,330,544	44.59%	98.24%	97.50%
N8V0-1	44,520,814	44.42%	97.24%	95.62%
N8V0-2	46,503,276	42.30%	97.39%	95.55%
N8V0-3	44,369,540	44.73%	97.23%	95.74%
N10V0-1	46,356,282	44.55%	97.39%	96.02%
N10V0-2	44,046,744	44.65%	97.56%	95.81%
N10V0-3	46,516,828	44.18%	97.40%	96.14%
N10V1-1	44,069,170	44.64%	98.18%	97.54%
N10V1-2	45,929,306	45.09%	98.39%	97.44%
N10V1-3	41,773,772	44.74%	98.25%	97.61%
N10V4-1	44,444,802	44.59%	97.97%	97.06%
N10V4-2	45,765,402	44.36%	97.91%	97.20%
N10V4-3	39,199,182	45.44%	98.39%	97.48%
N11V0-1	47,770,646	44.79%	97.55%	95.65%
N11V0-2	44,474,796	44.79%	98.29%	97.42%
N11V0-3	47,846,954	44.88%	98.08%	97.39%
N14V0-1	44,135,014	45.03%	98.28%	97.56%
N14V0-2	39,457,964	45.16%	98.39%	97.39%
N14V0-3	45,560,662	44.37%	98.22%	97.52%
Total	1,080,472,122			

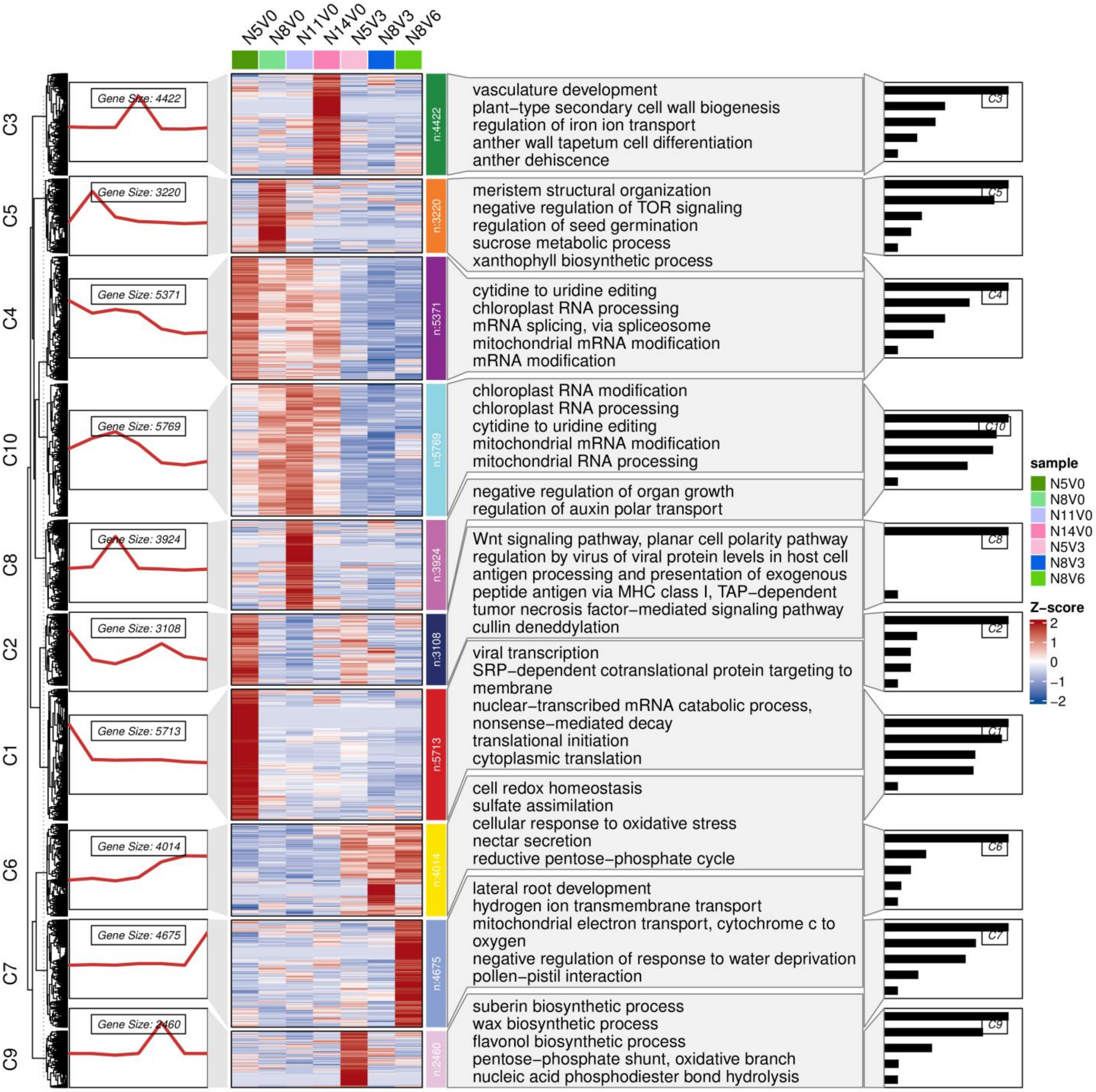

Figure S1. Time series analysis of dynamic gene expression changes during Group A vernalization. From left to right, cluster names, time series plots, all gene expression profiles, major GO enrichment processes (BP), and bar graphs are shown.
